# Supplementary material for: Asynchronous magnetic resonance elastography: Shear wave speed reconstruction using noise correlation of incoherent waves
Source: Magn Reson Med. 2022 Oct 27;89(3):990–1001. doi: 10.1002/mrm.29502 (PMC9792433; doi:10.1002/mrm.29502)
Supplement: Supplementary file 1 — DATA S1 MATLAB code used to generate the numerical simulations of Figure 1 [file MRM-89-990-s002.zip › k-Wave/helpfiles/example_na_optimising_performance.html]

Optimising k-Wave Performance Example (k-Wave)


# Optimising k-Wave Performance Example

This example demonstrates how to increase the computational performance of k-Wave using optional input parameters and data casting. A separate standardised benchmarking script `benchmark` is also included within the k-Wave toolbox to allow computational times to be compared across different computers and GPUs.

- Open the file in the MATLAB Editor
- Run the file in MATLAB

## Contents

- Controlling input options
- Data casting
- Running k-Wave on the GPU
- Multicore support
- C++ code

## Controlling input options

To investigate where the computational effort is spent during a k-Wave simulation, it is useful to use the inbuilt MATLAB profiler which examines the execution times for the various k-Wave and inbuilt functions. Running the profiler on a typical forward simulation using `kspaceFirstOrder2D` with a Cartesian sensor mask and no optional inputs gives the following command line output (set `example_number = 1` within the example m-file):

```
Running k-Wave simulation...
  start time: 04-Jun-2017 14:45:23
  reference sound speed: 1500m/s
  dt: 3.9063ns, t_end: 9.4258us, time steps: 2414
  input grid size: 512 by 512 grid points (10 by 10mm)
  maximum supported frequency: 38.4MHz
  smoothing p0 distribution...
  calculating Delaunay triangulation...
  precomputation completed in 1.7434s
  starting time loop...
  estimated simulation time 1min 3.1502s...
  simulation completed in 59.8023s
  total computation time 1min 1.61s
```

The corresponding profiler output is given below.

Aside from computations within the parent functions, it is clear the majority of the time is spent running `ifft2` and `fft2`. These are the fast Fourier transforms (FFTs) used to calculate spatial gradients as part of the k-space pseudospectral method used in k-Wave. Several seconds are also spent computing the Delaunay triangulation used for calculating the pressure over the Cartesian sensor mask using interpolation. The triangulation is calculated once during the precomputations and this time is encapsulated within the precomputation time printed to the command line. The Delaunay triangulation can be avoided by setting the optional input `'CartInterp'` to `'nearest'`, or by using a binary sensor mask. A Cartesian sensor mask can be converted to a binary mask using `cart2grid` as shown below.

```
% convert Cartesian sensor mask to binary mask
sensor.mask = cart2grid(kgrid, sensor.mask);
```

Several seconds are also spent running the various functions associated with the animated visualisation (`imagesc`, `newplot`, `cla`, etc). The visualisation can be switched off by setting the optional input `'PlotSim'` to `false`. Re-running the profile with these two changes gives the following command line output (set `example_number = 2` within the example m-file):

```
Running k-Wave simulation...
  start time: 04-Jun-2017 14:48:21
  reference sound speed: 1500m/s
  dt: 3.9063ns, t_end: 9.4258us, time steps: 2414
  input grid size: 512 by 512 grid points (10 by 10mm)
  maximum supported frequency: 38.4MHz
  smoothing p0 distribution...
  precomputation completed in 0.14281s
  starting time loop...
  estimated simulation time 56.6807s...
  simulation completed in 51.8237s
  total computation time 51.967s
```

The precomputation time has been reduced, and the loop computation time has also been reduced by several seconds. The corresponding profiler output is given below.


## Data casting

Even after the modifications above, the majority of the computational time is still spent computing the FFT and the point-wise multiplication of large matrices (within the function `kspaceFirstOrder2D`). It is possible to decrease this burden by capitalising on MATLAB's use of overloaded functions for different data types. For example, computing an FFT of a matrix of `single` type takes less time than for `double` (the standard data format used within MATLAB). For almost all simulations, the loss in precision as a result of performing calculations in `single` type is negligible, especially as the perfectly matched layer (PML) is only accurate to a few decimal points at best. Within `kspaceFirstOrder1D`, `kspaceFirstOrder2D`, and `kspaceFirstOrder3D`, the data type used for the variables within the time loop can be controlled via the optional input parameter `'DataCast'`. Re-running the profile with `'DataCast'` set to `'single'` gives the following command line output (set `example_number = 3` within the example m-file):

```
Running k-Wave simulation...
  start time: 04-Jun-2017 14:52:00
  reference sound speed: 1500m/s
  dt: 3.9063ns, t_end: 9.4258us, time steps: 2414
  input grid size: 512 by 512 grid points (10 by 10mm)
  maximum supported frequency: 38.4MHz
  smoothing p0 distribution...
  casting variables to single type...
  precomputation completed in 0.15796s
  starting time loop...
  estimated simulation time 37.7067s...
  simulation completed in 33.4639s
  total computation time 33.623s
```

The overall computational speed has been noticeably reduced. The corresponding profiler output is given below.


## Running k-Wave on the GPU

The computational time can be further improved by using other data types, in particular those which force program execution on a graphics processing unit (GPU). In particular, the MATLAB parallel computing toolbox contains overloaded MATLAB functions (such as the FFT) that work with any NVIDIA CUDA-enabled GPU. These toolboxes utilise an interface developed by NVIDIA called the CUDA SDK which allows programs written in C to run on the GPU, and then a MEX interface to allow the C programs to be run from MATLAB. Within MATLAB, the execution is as simple as casting the variables to the required data type. For example, to use the parallel computing toolbox within k-Wave, the optional input parameter `'DataCast'` is set to `'gpuArray-single'` or `'gpuArray-double'`.

To illustrate, the command line output obtained by setting `'DataCast'` to `'gpuArray-single'` is given below (set `example_number = 4` within the example m-file). For this particular hardware configuration, the computational speed is only slightly increased compared to setting `'DataCast'` to `'single'`, however, the improvement in performance becomes more pronounced for larger problems.

```
Running k-Wave simulation...
  start time: 04-Jun-2017 14:58:19
  reference sound speed: 1500m/s
  dt: 3.9063ns, t_end: 9.4258us, time steps: 2414
  input grid size: 512 by 512 grid points (10 by 10mm)
  maximum supported frequency: 38.4MHz
  smoothing p0 distribution...
  casting variables to gpuArray type...
  precomputation completed in 0.28512s
  starting time loop...
  estimated simulation time 25.8781s...
  GPU memory used: 1.0585 GB (of 6 GB)
  simulation completed in 23.8294s
  total computation time 24.117s
```

The corresponding profiler output is given below. The majority of time is now spent on computing matrix operations and the FFT on the GPU (within the function `kspaceFirstOrder2D`). Further details on the speed-up obtained when using different GPUs is given in `benchmark`.

  


## Multicore support

The command line and profile outputs shown here were generated using MATLAB R2013a. Some earlier MATLAB versions do not include multicore support for parallelisable functions such as the FFT. If using a very old version of MATLAB, it is possible to get a noticeable increase in computational speed simply by changing MATLAB versions.

## C++ code

For 3D problems and larger problems in 2D, a significant performance increase can be obtained by using the native C++ and CUDA versions of the simulation functions. See the Using the C++ Code examples, and the functions `kspaceFirstOrder2DC`, `kspaceFirstOrder2DG`, `kspaceFirstOrder3DC`, `kspaceFirstOrder3DG`, and `kspaceFirstOrderASC` for more details.
